# Books and Magazines

**Published:** 1899-05

**Authors:** 


### Books and Magazines.

---

HAY-FEVER AND ITS SUCCESSFUL TREATMENT. By W. C. Hallopeter, A. M., M. D., Clinical Professor of Pediatrics in the Medico-Chirurgical College, Philadelphia; Physician to the Methodist Episcopal Hospital; Fellow of the American Academy of Medicine, etc., etc. Published by P. Blakiston, Son & Co., Philadelphia. Pages, 135. Price, cloth, $1 net.

This is an excellent and complete monograph. All the details, historical, ætiological, pathological, are fully treated, and the therapeutics is especially full and up to date. An extensive bibliography shows that the author has made great research in its preparation.

Page(s) missing

He maintains that the disease is more amenable to treatment than is generally thought. I. J. J.

